# F25. NEURAPRO REVISITED: INCREASES IN LONG-CHAIN OMEGA-3 FATTY ACIDS IMPROVE FUNCTIONAL AND SYMPTOMATIC OUTCOMES IN ULTRAHIGH RISK PATIENTS

**DOI:** 10.1093/schbul/sby017.556

**Published:** 2018-04-01

**Authors:** G Paul Amminger, Barnaby Nelson, Hok Pan Yuen, Connie Markulev, Miriam R Schäfer, Monika Schlögelhofer, Nilufar Mossaheb, Stephan Smesny, Ian Hickie, Gregor Berger, Eric Chen, Lieuwe de Haan, Dorien H Nieman, Merete Nordentoft, Anita Riecher-Rössler, Swapna Verma, Maximus Berger, Andrew Thompson, Alison Yung, Patrick D McGorry

**Affiliations:** 1 Orygen, National Centre of Excellence in Youth Mental Health; 2 Medical University of Vienna; 3 University Hospital, Jena; 4 University of Sydney; 5 Child and Adolescent Psychiatric Service of the Canton of Zurich; 6 University of Hong Kong; 7 Academic Medical Center, Amsterdam; 8 Psychiatric Centre Bispebjerg; 9 Psychiatric University Clinics Basel; 10 Institute of Mental Health, Singapore; 11 Warwick Medical School; 12 Institute of Brain, Behaviour and Mental Health, University of Manchester

## Abstract

**Background:**

The NEURAPRO multicentre randomised controlled trial (RCT) of long-chain polyunsaturated omega-3 fatty acids (ω-3 PUFAs) (‘fish oil’) in combination with high-quality psychosocial intervention (cognitive behavioural case management [CBCM]) vs. placebo in combination with CBCM in young people at ultrahigh risk (UHR) of psychosis showed that the group allocated to fish oil had no clinical benefits over the placebo group. However, a limitation of the trial was that adherence with the study medication was relatively low. Furthermore, although RCTs are placed at the top of the evidence hierarchy, this methodology has limitations in fish oil RCTs, since the test agent is not only present in the intervention group, but ω-3 fats are present in the diet and in the tissues of all participants. A biomarker analysis of ω-3 changes during the trial can ultimately determine the efficacy of ω-3 supplementation in this trial.

**Methods:**

The NEURAPRO study was conducted from March 2010 to September 2014, in 10 specialized early psychosis treatment services in Australia, Asia, and Europe. In this study of 304 young people at UHR for psychotic disorders, 153 (50.3%) were allocated to ω-3 PUFAs and 151 (49.7%) to placebo. In all, 139 (45.7%) were male; mean (SD) age was 19.1 (4.6) years. The primary outcome was transition to psychosis assessed with the Comprehensive Assessment of the At-Risk Mental State. Secondary outcomes were levels of psychopathology and functioning assessed by the Brief Psychiatric Rating Scale (BPRS), the Scale for the Assessment of Negative Symptoms (SANS), the Montgomery Asberg Depression Rating Scale (MADRS), the Young Mania Rating Scale (YMRS), the Social and Occupational Functioning Assessment Scale (SOFAS), and the Global Functioning: Social and Role scales). Levels of ω-3 PUFAs in fish oil, eicosapentaenoic acid (EPA) and docosahexaenoic acid (DHA) (amongst other fatty acids) were measured as percentage of total fatty acids in erythrocytes at baseline and at month 6 (end-of-intervention). We examined changes in cell membrane levels of EPA and DHA, as measures of ω-3 intake independent of source. Data were analysed as a single cohort. Cox proportional hazards models and linear regression analyses were used to examine relationships between the ω-3 index (EPA+DHA) with clinical outcomes at month 6 and 12.

**Results:**

When analysed as a single cohort, no association was observed between the ω-3 index and transition to psychosis at any follow-up time point but increase of the ω-3 index was found significantly related with most of the functional and symptomatic measures at month 6 and 12, in linear regression models adjusting for relevant baseline factors (i.e., functioning, psychopathology, ω-3 index and smoking). The models revealed consistent results, with low functioning or high psychopathology at baseline, low levels of ω-3s at baseline and increase of the ω-3 index independently predicting clinical improvements at in this sample.

**Discussion:**

In contrast to our RCT analysis, this study using biomarkers shows that increase in erythrocyte ω-3 PUFAs may improve clinical outcomes of UHR patients. The results also imply that people with low DHA and EPA levels may benefit more from supplementation with fish oil. The analysis also highlights shortcomings of the RCT design in situations when the tested intervention is available outside the study.

